# The Representation of Foot and Leg Healing in Art: A Historical Iconographic Perspective from the Sixth Century Onward

**DOI:** 10.1007/s10943-025-02524-y

**Published:** 2025-12-15

**Authors:** Carlo Biz, Francesca Della Rocca, Alessandro Rossin, Luigi Pomes, Giuseppe Carolla, Michele Andreotti, Pietro Ruggieri

**Affiliations:** 1 Orthopedics and Orthopedic Oncology, Department of Surgery, Oncology and Gastroenterology DiSCOG, University of Padova, Via Giustiniani 3, 35128 Padua, Italy; 2 Art Historian (AH), Government Office for Archaeology, Fine Arts and Landscape, Folco Palace, Via Aquileia 7, 35139 Padua, Italy

**Keywords:** Anatomy, Art, Foot, History of medicine, Iconography, Medical illustration

## Abstract

This historical review aims not only to identify and analyse artistic depictions of miraculous healings of various injuries and orthopaedic pathologies affecting the foot and leg over the centuries, but also to explore the stories of the saints portrayed and the exceptional artists who brought these miracles to life through their masterpieces. By examining historical and artistic sources, this study traces the evolution of healing iconography from the earliest descriptions in the sixth century, through the Middle Ages to the Renaissance. Special attention is given to the increasing anatomical accuracy in these works of art, influenced by Renaissance figures such as Andreas Vesalius, who collaborated with artists like Titian. An iconographic analysis reveals the symbolic and didactic roles of these artworks in both religious and medical contexts. Findings reveal that religious art functioned both as a vehicle for theological messages and as an educational medical tool, strengthening the belief in divine healing and promoting new perspectives in the advances of active and dynamic medical science. These depictions supported the rise of church-affiliated hospitals, marking a transition from purely miraculous healing to practices grounded in anatomy and empirical medicine. This study highlights how depictions of foot and leg healing in religious art functioned simultaneously as expressions of faith and as vehicles for medical education for embracing new medical insights. This dynamic interplay between art, religion and medicine laid the foundation for the integration of visual representation into modern medical science, ultimately shaping the evolution of orthopaedics and anatomical understanding in subsequent centuries.

## Introduction

The first scientific treatises on medical subjects began to emerge in Europe from the thirteenth century onward, with early works primarily translated into Latin and later into the vernacular. These texts were influenced by the foundational knowledge passed down by ancient medical authorities, notably Hippocrates (b. ca. 460 BC on the Island of Kos—d. ca. 370 BC in Larissa) and Galen (b. ca. 130 in Pergamon—d. ca. 200 AD in Palermo), whose teachings formed the bedrock of Western Medical Science for centuries (Cosmacini, 2005; Grmek et al., 1998). The transmission of classical medical knowledge through translations played a pivotal role in shaping the medieval European medical landscape (Daly, 1999; Pesenti, 1985). At that time, despite the extensive attention given to organs located centrally within the body (heart, lungs and digestive system), early medical treatises devoted comparatively less focus to the lower extremities. This can be attributed to the foot’s more peripheral role in the body and its less immediate connection to life-sustaining functions (Crombie, 1959; QJM – Oxford Academic, 2016).

Nonetheless, over time, interest in the anatomical study of the lower limbs began to grow, particularly with the rise of Renaissance humanism, which emphasised the importance of detailed anatomical knowledge. Over the centuries, the foot and leg, studied from an anatomical, muscular and neurological perspective, began to receive greater attention, especially in artistic and medical representations, as their role in maintaining the upright posture and facilitating bipedal locomotion became more recognised (Biz et al., 2023a, 2023b; Ketham, 1493).

This shift in focus is evident in the works of Renaissance scholars and artists who sought to perfect the depiction of human anatomy, including the foot, which was now acknowledged for its complex mechanical functions. As the study of the foot began to evolve, the growing recognition of its importance in health and mobility reflected broader changes in medical understanding during this period (Fabrici d’Acquapendente, 1671; Zampieri, 2021; Marcon, 2004).

One of the major exponents of this scientific revolution of the Renaissance was Girolamo Fabrici d’Acquapendente (b. in 1533 in Acquapendente, Italy—d. in 1619 in Padua, Italy), an Italian anatomist, surgeon and physiologist, who built the oldest permanent anatomical theatre at Padua University, inaugurated in 1593, and still open to visitors today at the Palazzo del Bò (Vandenbulcke et al., 2020). He is also credited with the invention of the Oplomochlion, which is a collection of very diverse orthotic, prosthetic and surgical metal instruments used to treat several congenital and acquired deformities of the limbs and spinal column (congenital dislocation of the hip, congenital club-foot, rachitic deformities of the leg), and also to splint fractured limbs (Biz et al., 2019).

Sacred paintings, primarily designed to transmit universal, but exclusive religious messages to an illiterate and largely uninstructed populace, can be regarded as *“literature illiteratum”,* or *“the literature of analphabets”*. In the early Christian era, as the Church gradually asserted dominance over the remnants of pagan religions, it absorbed many old practices into its rituals. Pagan gods, with their distinctive attributes, were replaced by various saints and their corresponding symbols. This transition allowed the functions of the former deities to be carried on under the guise of Christian saints.

This process of incorporating Christian legends began early in the Church’s history, and by the fourth and fifth centuries, the Byzantine culture had established a visual tradition for the sacred story. This tradition was accepted as both authoritative and foundational for religious art. Artists and craftsmen who interpreted these sacred stories followed a well-defined path, guided by the narratives of the Old and New Testaments, in particular the miracles of Jesus Christ and the Apostles (Mathews, 1999).

Throughout the centuries, in the Christian tradition, the saints have provided hope and comfort to patients. They have also served as inspirational figures for physicians facing limited resources, offering both spiritual support and role models in the face of adversity (Lasso et al., 2022). The lives and legends of these saints offer invaluable insight into the medical culture of past centuries, reflecting the intersection of faith and healing during times when medical knowledge was still in its infancy (Williams et al., 2022).

Among the numerous Christian figures with connections to medicine, the most popular are St. Luke, St. Benedict, St. Lucy, St. Sebastian, St. Roch and St. Agatha; however, only a few are generally or comprehensively associated with and specialised in trauma and orthopaedic conditions. St. Ignatius of Loyola (b. on 23 October 1491 in Loyola, Spain—d. on 31 July 1556 in Rome, Italy), patron saint of trauma and fracture surgery, stands out for his pioneering efforts in deformity-correction surgery, ironically performed on himself. St. Kostka (b. on 28 October 1550 in Zembów, Poland—d. on 15 August 1568, in Rome, Italy), the patron saint of broken bones, is renowned for enduring immense suffering at the hands of his notoriously cruel brother Paul. St. Alphonsus Liguori (b. on 27 September 1696 in Marianella, Italy—d. on 1 August 1787 in Nocera de’ Pagani, Italy), who is the patron saint of spinal conditions, tragically suffered from such a severe cervical kyphosis that his chin progressively eroded his chest. Other saints included in this group are St. Amalberga (b. ca. 741 in Ardennes, Belgium—d. on 10 July 772 in Temse, Belgium) and St. Roch (b. ca. 1345 in Montpellier, France—d. ca. 1376 in Voghera, Italy), who are associated with upper and lower limb and knee injuries, respectively (Gulisano, 2011).

Particularly within the Dominican tradition, the healing of the foot, among other miraculous acts, is also attributed to Saints Cosmas and Damian (b. ca. 260 in Laiazzo—d. ca. 303 in Cyrrhus, Antioch), as well as to Saint Peter Martyr of Verona (b. on 29 October 1205 in Verona, Italy—d. on 6 April 1252 in Milan, Italy), a key figure in the Dominican Order. Similar miracles are associated with Saint Francis of Assisi (b. in 1181–1182—d. on 3 October 1226, in Assisi, Italy) and Saint Anthony of Padua (b. on 15 August 1195 in Lisbon, Portugal—d. on 13 June 1231 in Padua, Italy), important figures of the Franciscan Order.

Among the miracles recorded in the lives of the Saints, the iconography of foot reattachment becomes codified from the fifteenth century onward, when Bartolomeo and Agnolo degli Erri, Donato of Niccolò of Betto Bardi (called *Donatello*) in 1446, Lorentino d’Andrea in 1452–1466 and Vincenzo Foppa in 1468 were commissioned to represent in vivid detail the miraculous reattachment of a foot performed by a saintly healer and attributed to Saint Anthony of Padua and to Saint Peter Martyr of Verona (Rinaldi, 1995). The different attributions of the same miracle can be explained by the historical competition between the Franciscan Order, to which Saint Anthony belonged, and the Dominican Order, to which Saint Peter belonged, as both were credited sacred healings.

The aims of this historical review are twofold: first, to identify and analyse artistic representations of miraculous healings of various injuries and orthopaedic pathologies affecting the foot and leg, dating from the sixth century onward; and second, to explore the lives of the depicted saints as well as the extraordinary artists responsible for these figurative representations that hold particular relevance to the early history of orthopaedics.

## Rise and Walk: Lower Limb Healings Attributed to the Religious Figures of the Gospels and the Acts of the Apostles

Alongside the early scientific interest in medical manuscripts and printed texts, some of the earliest visual depictions of foot healing are attributed to Jesus Christ, the Virgin Mary, the Disciples and the Apostles. The healing of a paralytic by Jesus is described in the Gospel of Matthew 9:1–8, Mark 2:1–12, Luke 5:17–26 and John 5:1–9. In the Acts of the Apostles, the healing of a crippled man by the Apostles Peter and John in the name of Jesus 3:1–10 is reported, as are two other similar healings by Peter 5:12–14 and 9:32–35. A foundational biblical narrative in this context is the healing of the lame man at the Beautiful Gate, described in Acts of the Apostles 3:6–8: *“Peter said: Silver or gold I do not have, but what I do have I give you. In the name of Jesus Christ of Nazareth, walk. Taking him by the right hand, he helped him up, and instantly the man’s feet and ankles became strong. He jumped to his feet and began to walk”* (The text of Gospels and Acts of Apostles are from New Testament).

This episode, which emphasises the miraculous strengthening of feet and ankles, established a symbolic association between healing and the lower limbs in Christian iconography. Peter’s healing of the lame man holds particular significance. First, it recalls the many instances in which Jesus healed crippled individuals, such as the paralytic. Second, the man at the temple gate was known to be lame from birth; he had never walked, underscoring the miracle’s authenticity and gravity. Most importantly, Peter’s act fulfils a key Messianic prophecy: *“Then will the lame leap like a deer”* (*Isaiah 35:6*) (The Holy Bible. Book of Isaiah, Chapter 35, verse 6). This vision of a transformed world where infirmity is reversed and divine presence brings renewal directly links the ministry of Jesus to that of his apostles. These powers of the Apostles stirred envy in Simon Magus, a first-century AD religious figure, who dazzled people of Samaria with trickery and sorcery and was then converted to Christianity by the Evangelist Philip. He offered to pay Peter in exchange to learn this power, inciting Peter’s wrath. *“‘Give me also this ability so that everyone on whom I lay my hands may receive the Holy Spirit.’”But Peter answered: “‘May your money perish with you, because you thought you could buy the gift of God with money!” ‘* (Acts of the Apostles, Chapter 8: 9–10; 19–20). This episode gave rise to the term ‘simony’, to describe the act of selling church offices and roles or sacred things (Knowles, 2005).

In contrast to the healings of the lower limbs, when Simon was performing magic in the Forum and seeking to prove himself to be a god, he levitated into the air. The apostle Peter prayed to God to stop his flying. Simon stopped in mid-air and fell, breaking his ‘anklebone’ (Connolly, 1969), or more precisely, his *‘astragalo’* (talus) (Biz et al., 2023a, 2023b; Ragucci, 2013).

**Fig. 1:**
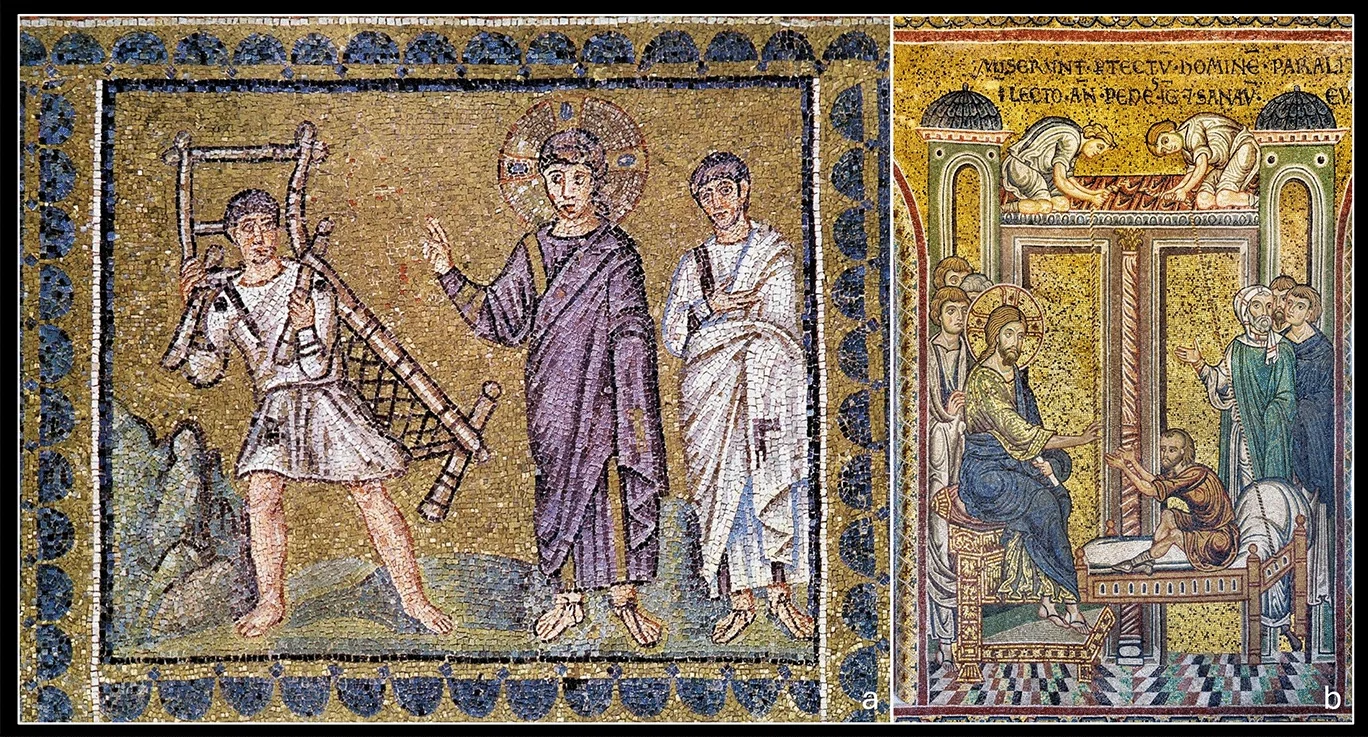
**a** Mosaic depicting *a paralytic man carrying a stretcher,* symbolising helplessness, sixth century A.D., from the Church of San Apollinare Nuovo in Ravenna, Italy. **b** Mosaic showing a *paralytic man rising from his bed,* thirteenth century, located on the wall of the Cathedral of Monreale (Palermo), Italy

Earlier artistic depictions illustrate miraculous healing of the lower limbs, most of which are of anonymous patients. A paralytic who carries a stretcher rendered useless is depicted in the 6th-century mosaic on the wall of the *Church of New Saint Apollinaire in Ravenna* (Fig. 1a). In the dome fresco of the *Baptistery of Saint John the Baptist in Parma,* painted shortly after its consecration in 1216, two leprosy-afflicted individuals are shown having lost their feet (Morlacchi, 1997, card 147, pages 252–253). A paralytic who gets up from his bed is depicted in a 13th-century mosaic on the wall of the *Cathedral of Monreale in Palermo* (Fig. 1b). Between 1330 and 1336, Andrea Pisano (b. in 1295 in Pisa—d. ca.1348 in Orvieto, Italy) sculpted scenes from the lives of Saint John the Baptist and Jesus on bas-relief of the south doors of the *Baptistery of Florence*, including a square with two crippled figures awaiting healing (Morlacchi, 1997).

**Fig. 2:**
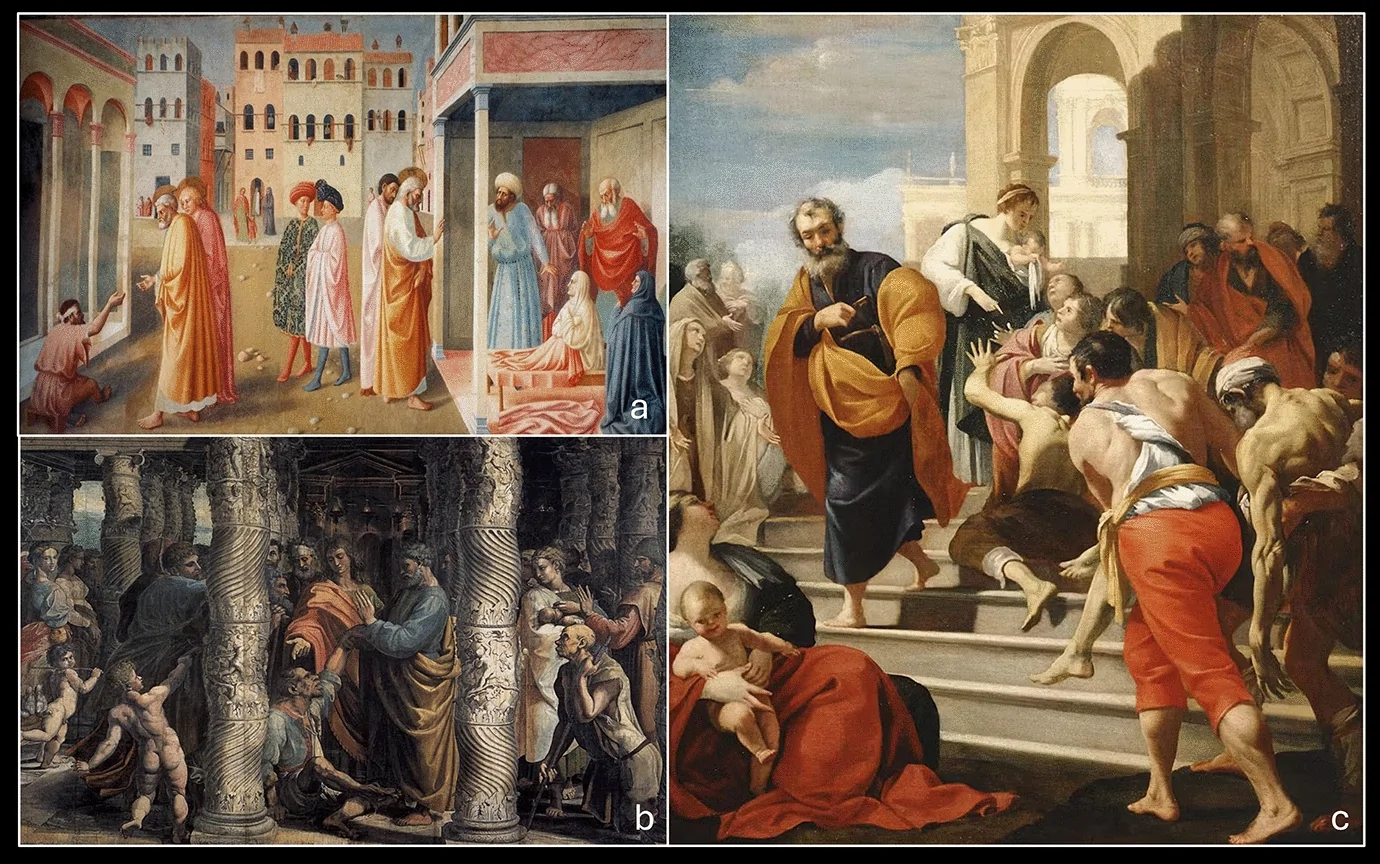
**a** Masolino da Panicale, *Saint Peter Healing the Lame Man*, 1424, Brancacci Chapel, Church of Santa Maria del Carmine, Florence, Italy. **b** Raffaello Sanzio (Raphael), *The Healing of the Lame Man*, cartoon for a tapestry, ca. 1515–1516, Victoria and Albert Museum, London, UK. **c** Laurent de La Hyre, *The Healing of the Lame Man*, ca. 1650, Uffizi Gallery, Florence, Italy

This biblical legacy found compelling expressions in Renaissance art. In 1424, in the *Brancacci Chapel of the Church of Santa Maria del Carmine in Florence*, Masolino da Panicale (b. in 1383 in Panicale—d. in 1440 in Florence, Italy) depicted *Saint Peter healing the lame man* (Fig. 2a) (Hartt & Wilkins, 2012). Nearby, Tommaso of Ser Giovanni of Mone Cassai, called *“Masaccio”* (b. in 1400 in Arezzo—d. in 1428 in Rome, Italy), portrayed the same apostle healing a man afflicted with poliomyelitis using only the power of his shadow, rendered with remarkable realism and psychological depth (Freedberg, 1993). These frescoes not only reflect theological teachings but also demonstrate an evolving visual language for representing divine intervention in the physical body.

Among the most significant examples are works painted by renowned Renaissance artists such as: Raphael (b. in 1483, Urbino—d. on 6 April 1520 in Rome, Italy), Tintoretto (b. in 1518—d. in 1594 in Venice, Italy), Jacopo Palma il Giovane (b. in 1544—d. in 1628 in Venice, Italy), Laurent de La Hyre (b. on 27 February 1606—d. on 28 December 1656 in Paris, France) and Nicolas Poussin (b. on 15 June 1594 in Les Andelys, France—d. on 19 November 1665 in Rome, Italy) (Brown, 2009). One notable series is the *Raphael Cartoons*, a full-scale tapestry design for the Sistine Chapel commissioned by Pope Leo X shortly after his election in 1513. These monumental compositions depict key episodes from the lives of Saints Peter and Paul, foundational figures of the early Church (Fig. 2b) (Paoletti & Radke, 2005). Among them, the scene of the healing of the crippled man by the Apostle Peter reflects the painter’s commitment to realism, possibly inspired by the many crippled and lame individuals who frequented churches during his time. The same evidence is found in the masterpiece of Laurent de La Hyre, purchased in Paris in 1793 and immediately brought to Florence, now housed in the Uffizi Gallery (Fig. 2c) (Callot, 1619).

In the early seventeenth century, the engraver Jacques Callot realised the drawing by Matteo Rosselli and for the *Servite friar* Giovanni Angelo Lottini of a miraculous healing attributed to the Virgin Mary of a sick man named Leonardo di Massa di Cassara afflicted by the *“storpiate membra”* who went to the *Church of the Most Holy Annunciation in Florence* for healing. This masterpiece is interesting because it testifies to the name and location of the patient, who was healed in a church near the famous *Hospital of the Innocents,* which was completed by 1417 (Callot, 1619) (Jacques Callot from Matteo Rosselli, 1619). The link between church and hospital became very strong from the beginning of the fifteenth century. For example, in Padua, the nursery of anatomical studies, Saint Francis’ Hospital was built in the shadow of the cloister of the eponymous church (Ricalde, 2015).

A miraculous event that occurred in 1640 in Calanda, a town in Aragón, Spain, was the subject of a book, *Il miracolo* by Victor Messori (Rizzoli, 1998). The event provides an interesting basis for clinical deductions related to limb replantation (Messori, 1998). This time, the miraculous healing happened through the intercession of the Virgin Mary, the mother of Jesus. In 1638, the 21-year-old Juan Pellicer from Calanda had his right leg amputated at the Hospital of Zaragoza (Cugola, 2000), following an agricultural accident. In March 1640, one night, Juan’s parents were astonished to find that their son, while sleeping, had two legs. A miraculous replantation had occurred. One can imagine the “stir” caused by this event, historically documented through the original notarial records and a ruling from the Archbishop of Zaragoza after a trial was held to verify the “validity” of the occurrence. The jurors were canonical representatives and professors from the University of Zaragoza. It is worth noting that Juan Pellicer was a devout follower of the *Virgin of Pilar*. This is the only documented miraculous replantation in history, although many other instances of replantation of arms and legs are recorded in Christian tradition, supported by a rich body of iconographic evidence (Reynaud, 2010).

## The Miracle of the Black Leg

The earliest and most renowned saints associated with miraculous foot transplantation are the twin physician-saints, Cosmas and Damian, patrons of medicine and surgery, musculoskeletal oncology, dentists, and pharmacists, as well as barbers, the ancient “cerusici”, the surgeons of the past (Hernigou, 2014). Many pharmacies are dedicated to Cosmas and Damian; for example, one can be found in the Giudecca district of Venice. The Catholic Church used to celebrate the liturgical feast for them on September 27 (probably the day of the dedication of the Roman basilica), but Pope Paul VI moved the celebration to September 26, making the devotion optional. The Orthodox Church commemorates them on July 1, October 17, and November 1, following three different traditions: Roman, Arabic and Asiatic.

Born in 3rd-century Arabia, the brothers studied in Antioch and practised medicine in Aegeae, a coastal town in the bay of Alexandretta, modern-day Turkey, where they healed and cured both humans and animals. Known for their refusal to accept payment, they earned the epithet Anargyroi, or *“the Silverless Ones”.* Under the reign of the Emperor Diocletian (AD 284–305), they were forced to renounce their faith. Their medical and spiritual legacy remains deeply embedded in Christian tradition and iconography.

Their most celebrated miracle is the Miracle of the Black Leg, a feat widely recognised as the first recorded homoplastic limb transplant. According to the 13th-century “*Legenda Aurea”* by Jacopo da Varagine (b. in 1230 in Varazze, Savona—d. in 1298 in Genoa, Italy), Deacon Justinian of Rome suffered from a grave disease of the leg, described variously as a cancer or tumour. In a dream, Cosmas and Damian appeared to him, amputated the gangrenous limb and replaced it with the healthy leg of a recently deceased Ethiopian man. The deacon made a full recovery, and the event came to be known by the distinctive colouration of the transplanted limb (Jacopo da Varagine, 1900a, 1900b).

**Fig. 3:**
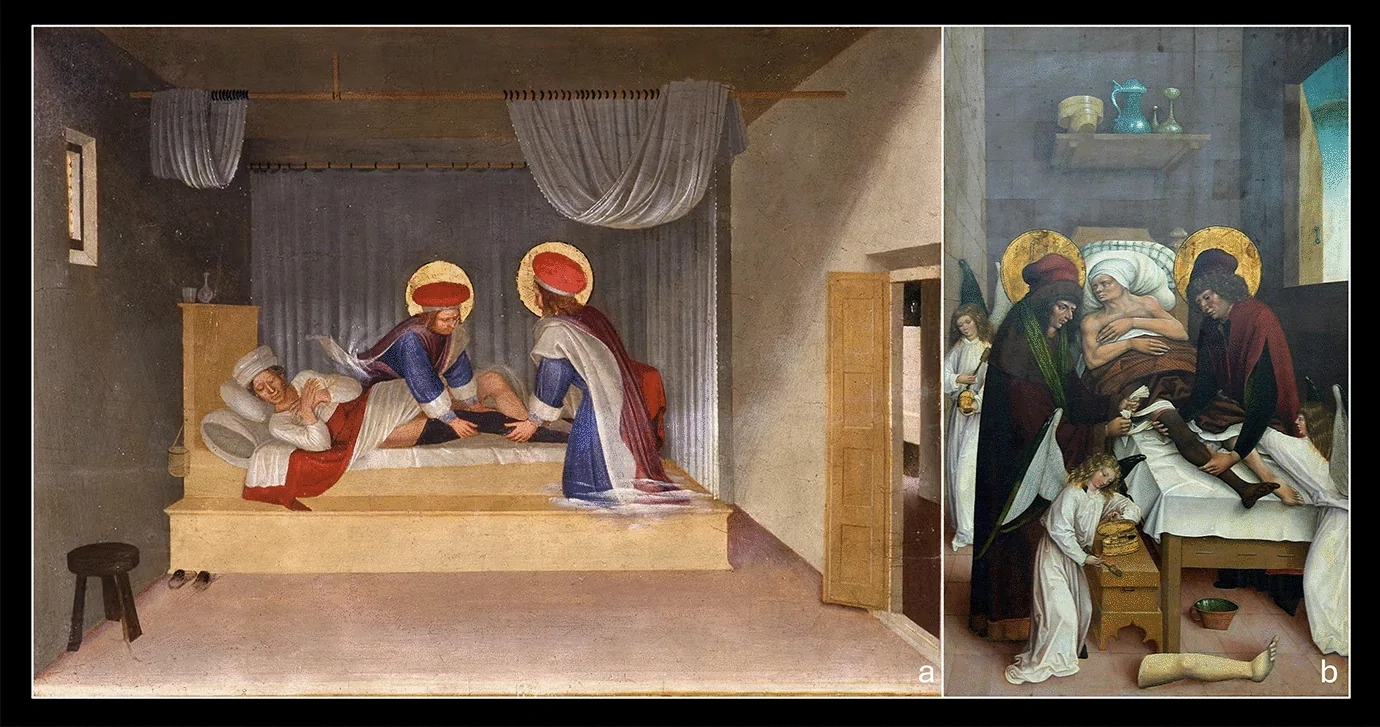
**a** Guido di Pietro, known as Fra Giovanni da Fiesole (Beato Angelico), *The Transplantation of the Black Leg*, ca. 1438, San Marco Museum, Florence, Italy. **b** Anonymous artist, German School, *The Transplantation of the Black Leg*, ca. 1515, Landesmuseum Württemberg, Stuttgart (Stockard) Germany

This miracle was first vividly captured in 1438–1443 by Dominican friar and painter Guido di Pietro or Fra Giovanni da Fiesole (b. ca. 1395—d. in 1455, in Rome, Italy), better known as *Beato Angelico*, in a predella panel for the Convent of San Domenico in Cortona (in the province of Arezzo), now preserved in the San Marco Museum in Florence, where Cosmas and Damian were patrons of the Medici family (Fig. 3a). The painting not only illustrates the miraculous procedure but also symbolically bridges early surgical imagination with divine intervention (Hartt, 2010).

Despite their healing legacy, the brothers’ Christian faith drew the ire of Emperor Diocletian. They were subjected to brutal tortures, such as crucifixion, stoning and drowning. They were ultimately beheaded in 303 AD (Eusebius, 1965). Their bodies were taken to Cyrrhus in northern Syria and were buried at the site of an ancient temple dedicated to the Greek god Asklepios, where the Priests treated patients by incubation (Scarborough, 1985). Their martyrdom only amplified their veneration. Pope Felix IV (his pontificate 526–530 AD) later transformed the ancient Library of Peace in the Roman Forum into a basilica bearing their names, incorporating structures from Emperor Maxentius and commissioning iconic mosaics depicting the saints led to Christ by St. Peter and St. Paul (Krautheimer, 1986).

Artistic interpretation of their miracles flourished across Christendom, especially during the Renaissance. Beyond *Fra Angelico’s Cortona cycle*, panels depicting their martyrdom and healing works appear in collections in Munich, Dublin, Paris and Florence. For example, the predella of the altarpiece of Cosmas and Damian by Sano di Pietro (b. in 1405 in Siena, Italy—d. in 1481) depicts the moment of the amputation from the black man just after he died (Bacchi, 1997). For a better understanding of the legend’s content, some Northern Renaissance artists illustrated the diseased leg beside the patient’s bed just after it was removed from the body (Fig. 3b). These works of art, rendered with a blend of narrative clarity and spiritual simplicity, underscore their role not just as healers of physical bodies but of faith (Campbell, 2001).

Their story reflects the medieval fusion of medicine, faith and imagination. Long before the advent of scientific transplantology, the Miracle of the Black Leg foreshadowed modern concepts of donor–recipient dynamics. It finds an echo in the early 20th-century work of Alexis Carrel, whose breakthroughs in vascular anastomosis earned him the Nobel Prize in 1906 (Hernigou, 2014; van Gulik, 2024). In an era when disease and injury instilled both fear and faith, the holy surgeons Saint Cosmas and Saint Damian offered a supernatural answer to human frailty, leaving a legacy that bridged the boundaries among theology, medicine and art.

## The Healings of the Lower Limbs by Saint Francis of Assisi

The miracle of the replanting of a severed foot is famously attributed to Saint Francis of Assisi (b. 1182 and d. 1226 in Assisi), the “poor man of Assisi”, the “jester of God”, who founded the Franciscan Order in 1209 and who was proclaimed Patron of Italy in 1939 (together with Saint Catherine of Siena) by Pope Pio XII (Johnson, 2009). He was canonised in 1228, just a couple of years after his death; his feast day is celebrated on October 4. Saint Francis of Assisi is the patron saint of people of many professions, including upholsterers, merchants, students, philosophers and environmentalists. Venice has dedicated an island to him, *Saint Francis of the Desert*, commemorating his legendary passage through the lagoon in 1220. He is credited with the creation of the nativity scene. On Christmas night in 1223, in Greccio, the saint reenacted the birth of Jesus by creating a living representation (Cook, 2005).

Saint Francis was born in 1182 to Pietro Bernardone dei Moriconi and the noblewoman Pica Bourlemont, of French origin, in a wealthy family that traded in Provence. His mother had him baptised with the name Giovanni in the church built in honour of the city’s patron saint, the martyr Rufinus. However, his father decided to change his name to Francis, which was unusual for the time, in honour of France, where he had made his fortune. After attending school with the canons of the Cathedral, at the age of 14, Francis threw himself into commerce, without neglecting cheerful outings with friends. Then everything changed: he was involved in the wars between the Guelphs and Ghibellines and ended up imprisoned in Perugia. The experience shocked him and marked the beginning of his conversion journey. Back home, he spent much time on his father’s properties. According to Thomas of Celano, it was in those quiet places that a deep love for nature awakened in him, allowing him to see nature as a marvellous work of God (Celano, 1999a, 1999b). In 1203, he decided to join the Crusades and attempted to reach Lecce, where Count Walter III of Brienne was gathering forces, to head to Jerusalem with other knights. Upon reaching Spoleto, he fell ill and turned back. It is said that one day, in the Church of San Damiano, he heard the Crucifix speak to him, saying three times: “Francis, go and repair my house, which, as you see, is falling into ruin.” (Celano, 1999a, 1999b).

The First Life of the Saint, written by Thomas of Celano (b. in 1190—d. in 1260 in Aquila, Italy) between 1228 and early 1229, is the first biography of the Saint of Assisi. This became the foundation for several other Lives or Legends that repeat its structure. Giuliano da Spira (b. ca. 1190 in Speyer, Germany—d. ca. 1250, likely in Italy), a German Franciscan and one of the most active followers of Francis, wrote *Life of Saint Francis* as well as a biography of Saint Anthony. Another treatise is the “*Legenda Major*”, written in 1263 by Bonaventura da Bagnoregio (b. in 1221—d. in 1274 in Lyon, France), the successor of Saint Francis as the chief of the Franciscan Order. It was the official source for most of the artistic representations, including the most famous Life of the Saint, frescoed in the Church dedicated to Saint Francis in Assisi (Bonaventure, 1999).

Most of the miracles reported in the Legenda Major are related to the lower limb healings: “*in the city of Toscanella, he [Francis] was devoutly entertained as guest by a certain Knight, whose only son was crippled from birth; at his own urgent entreaty, he raised him with his hands, and so suddenly made him whole that, in the sight of all, his limbs were all forthwith strengthened, and the boy, made whole and strong, rose up at once, walking and leaping and praising God”* (Bonaventura from Bagnoregio, 1263a, 1263b, 1263c, 1263d)*.*

*“In the city of Narni, when, at the entreaty of the bishop, he [Francis] had made the sign of the Cross from head to foot over a certain paralytic who had lost the use of all his limbs, he restored him perfectly to health*” (Bonaventura from Bagnoregio, 1263a, 1263b, 1263c, 1263d)*.*

“*In the diocese of Rieti, a boy that from the age of four had been so swollen that he could in no wise look on his own legs, was brought unto him [Francis] by his mother with tears, and forthwith, when the Saint touched him with his holy hands, was healed*” (Bonaventura from Bagnoregio, 1263a, 1263b, 1263c, 1263d)*.*

“*In the city of Orte, a boy was so deformed that his head rested on his feet, and some of his bones were broken; he, when Francis at the tearful entreaties of his parents had made the sign of the Cross over him, on a sudden stood upright, and was from that moment unloosed*” (Bonaventura from Bagnoregio, 1263a, 1263b, 1263c, 1263d)*.*

Thomas of Celano reported the healing of Narni and refers to “the certain paralytic” as a boy with more medical details: *“In the territory of Narni, there lived a boy with such a deformed tibia that he could only move by leaning on two crutches. He was poor and lived on alms, as he had been ill for many years and didn’t even know his father and mother. Through the merits of our most blessed father Francis, he regained full health and walked freely without the crutches, praising and blessing God and His faithful servant”* (Tommaso from Celano, 1228).

**Fig. 4:**
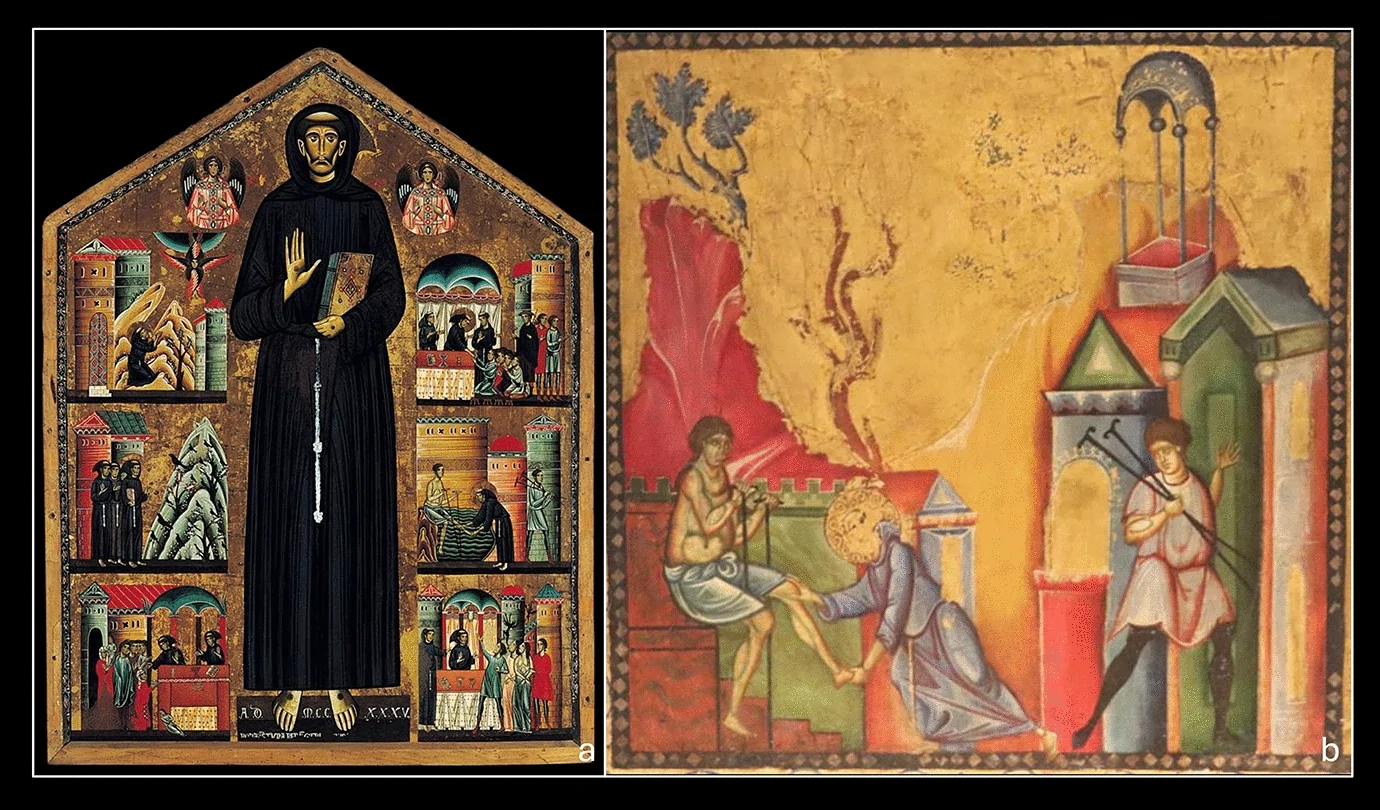
**a** Bonaventura Berlinghieri, *The Healing of the Tibia by Saint Francis (detail from the Table of the Stories of Saint Francis)*, 1235, Church of Saint Francis, Pescia (Pistoia), Italy. **b** Attributed to Giunta Pisano, *Saint Francis and Four Post-Mortem Miracles*, ca. 1250–1260, Sacred Museum, Assisi, Italy

The healing described by Bonaventura da Bagnoregio and Tommaso da Celano is faithfully represented in one of the *“Stories of the Life of Saint Francis”* painted by various artists in the thirteenth century, in paintings called “hagiographic tables” suitable to show the life and miracles of the Saints in a sequence of boxes surrounding the frontal portrait of the Saint. Among them, the painters known as the Master of the Cross no. 434 (from ca. 1230—half thirteenth century) and Master of Santa Maria Primerana (born about 1220—died ca. 1270), Bonaventura Berlinghieri or Berlinghiero from Lucca (flourished 1228—between 1236 and 1242) and Giunta Pisano or Giunta Capitini (active in Pisa from 1202 to 1236) painted the miraculous intervention of the Saint, by which the boy regains the use of his legs and lifts the now-useless crutches with one arm (Boskovits, 1988). The more pictorially accurate is the masterpiece painted in 1235 by Berlinghieri, the first representation of the Saint, painted only nine years after his death. It is very precise in the portrait of the young boy and the healing Saint (Fig. 4a and b).

## Saint Anthony of Padua and the Attaching of the Cut-Off Foot

Viewed from an ethnic-medical perspective, Anthony of Padua (b. 15 August 1195 in Lisbon—d. June 13, 1231 in Padua) is the most popular and venerated Christian saint in the world. He is primarily venerated as the patron saint of young children, the protector against sterility, the helper of women in labour, women seeking a husband, the poor and the hungry, those who have lost things, as well as those suffering from fever, dropsy and eye diseases. No less interesting are the numerous legends of miracles attributed to the Saint’s charismatic powers, particularly those related to his compelling sermons.

Saint Anthony of Padua, born into a noble family as Fernando Martins de Bulhoes y Taveira de Azevedo, began his religious life with the Augustinians but joined the Franciscans after being inspired by the martyrdom of five friars. Taking the name Anthony, he attempted missionary work but fell ill and was shipwrecked in Sicily. After recovering, he travelled to Assisi and eventually lived in Montepaolo in profound humility before becoming a renowned preacher. He fought heresy across northern Italy and France, earning the title “Hammer of Heretics”. A skilled theologian, he was later appointed provincial minister of Northern Italy and settled in Padua, where he defended the poor and opposed tyranny. Known for his wisdom and powerful sermons, which were reportedly understood in multiple languages simultaneously, he became a trusted voice of the Church. In declining health, Anthony retired to Camposampiero but asked to be brought back to Padua as he sensed his death nearing. He died on 13 June 1231 in the convent of Arcella (Spilsbury, 2010). News of his death spread quickly, with children in the city reportedly shouting, *“The Saint is dead”.*

He was canonised in May 1232, just a year after his death, and his feast day is celebrated on June 13. He was proclaimed a Doctor of the Church in 1946 and is said to have performed miracles in life and after death (Severn, 2019). His miracles inspired many artists, including Simone Martini, Titian and Donatello, who depicted him with a white lily (*a symbol of purity*) and the baby Jesus in his arms (Williams et al., 2022).

The most relevant story is that of young Leonard from Padua, who confessed to Anthony that he had struck his mother with his foot. Saddened by this fact, Anthony allegedly commented bitterly, *“The foot that strikes its parents should be cut off”.* Depressed, Leonard returned home and, in despair, sawed off his foot. Upon witnessing this, his terrified mother cried out for help. Suddenly, Anthony (the future saint) appeared out of nowhere and, miraculously, reattached the severed foot to the boy’s leg (Meagher, 2002; Perciaccante et al., 2016).

Among the various lives of the Saint, the first ones are the *“Vita Prima”* or “*Assidua*” and the *“Vita Secunda”*, written, respectively, in about 1232 and about 1235, the first by an anonymous Franciscan friar and the second by Friar Julian of Spira, who also wrote one of the Lives of Saint Francis. They do not describe any miracles performed by the Saint during his lifetime. Rather, they recount healings that occurred after his death, through his saving intercession, most likely carried out by friar-medics-healers acting in his name (Gamboso, 1986a, 1986b). It is interesting that the anonymous Franciscan friar, author of the *Dialogus,* dated 1245-46, just over a decade after the Saint’s death, records in meticulous detail, for each patient, their baptismal name, the place of healing and the illness from which they suffered, almost like a medical logbook (Gamboso, 1986a, 1986b). Among the most common ailments, those affecting the lower limbs play a significant role, likely due to their importance in performing many vital functions, especially for those who, in their work, had to rely primarily on their physical abilities.

**Fig. 5:**
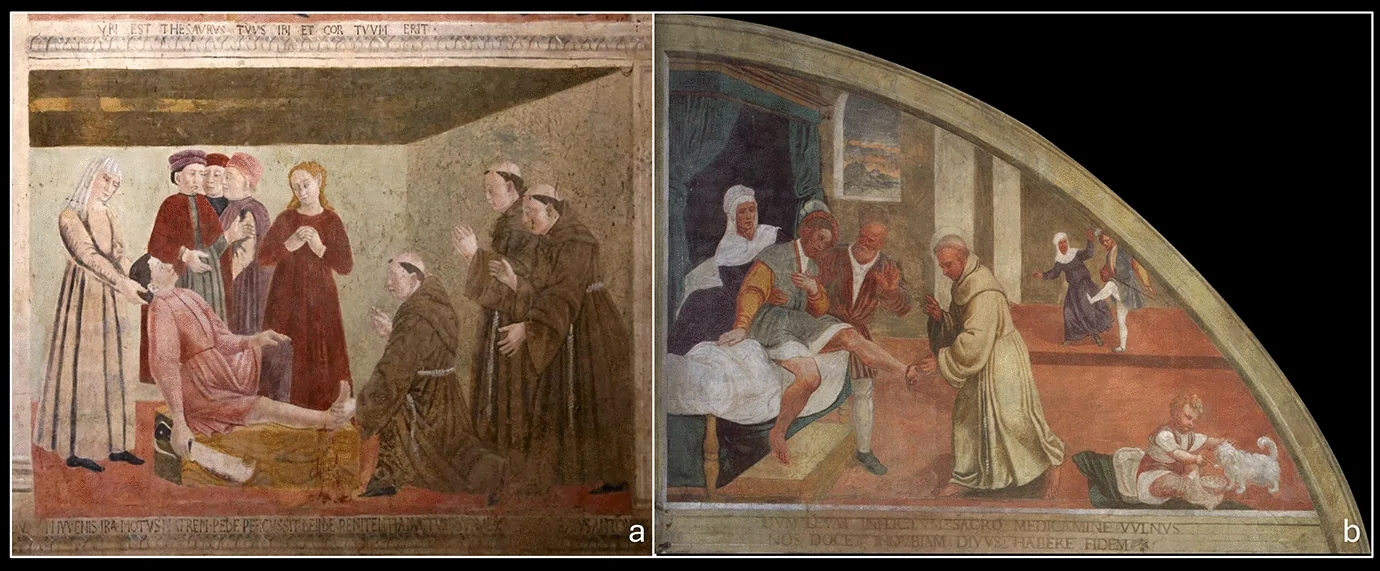
**a** Lorentino d’Andrea, *The Reattachment of the Severed Foot by Saint Anthony of Padua,* 1452–1466, Church of Saint Francis, Arezzo, Italy. **b** Girolamo Tessari (known as Dal Santo), *The Reattachment of the Severed Foot by Saint Anthony of Padua,* 1535, Santuario del Noce, Camposampiero (Padova), Italy

Scenes of replantation play a central role in the *Church of San Francesco in Arezzo* (1452–1466). In the *chapel of Saint Anthony of Padua*, Lorentino d’Andrea (b. in Arezzo ca. 1430—d. 1506), working alongside Piero di Benedetto de’ Franceschi, known as Piero della Francesca (Sansepolcro 1412–1492), depicts the healing scene, explaining the miraculous healing with the caption: *“[…] a young man, moved by anger, struck his mother with his foot, then, overcome by penitence […] Anthony healed him” *(Fig. 5a).

**Fig. 6:**
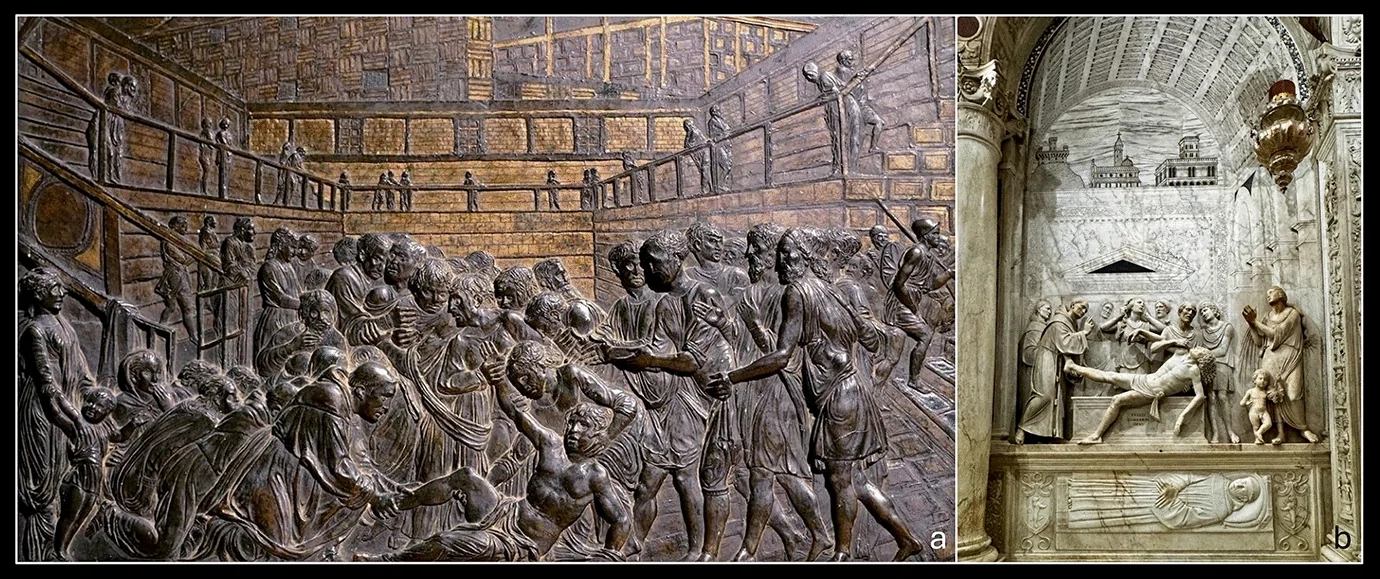
**a** Donato di Niccolò di Betto Bardi (known as Donatello), *The Reattachment of the Severed Foot by Saint Anthony of Padua*, 1446, Altar of the Saint, Basilica of Saint Anthony, Padua, Italy. **b** Tullio Lombardo, *The Reattachment of the Severed Foot by Saint Anthony of Padua*, 1504, fifth arch on the right of the back wall, Ark of the Saint, Basilica of Saint Anthony, Padua, Italy

In Padua, the confrères of the *Veneranda Arca del Santo* chose to emphasise the miracles of Saint Anthony of Padua, with particular focus on the attaching and healing of the cut-off foot. This motif is repeatedly featured in key sites of devotion, including the Altar and the Ark dedicated to the miraculous Saint, as well as the ambulatory and the choir of the Basilica of Saint Anthony, which was restored after the fire of 1394. Around 1446, this event was depicted by Donato di Niccolò di Betto Bardi, known as *Donatello* (b. in 1386—d. on 13th December 1466 in Florence, Italy), in one of four bas-reliefs illustrating the Saint’s miracles that decorate the Altar (Fig. 6a), today known according to the later reconstruction by Camillo Boito (1895), Gasparotto (1975).

**Fig. 7:**
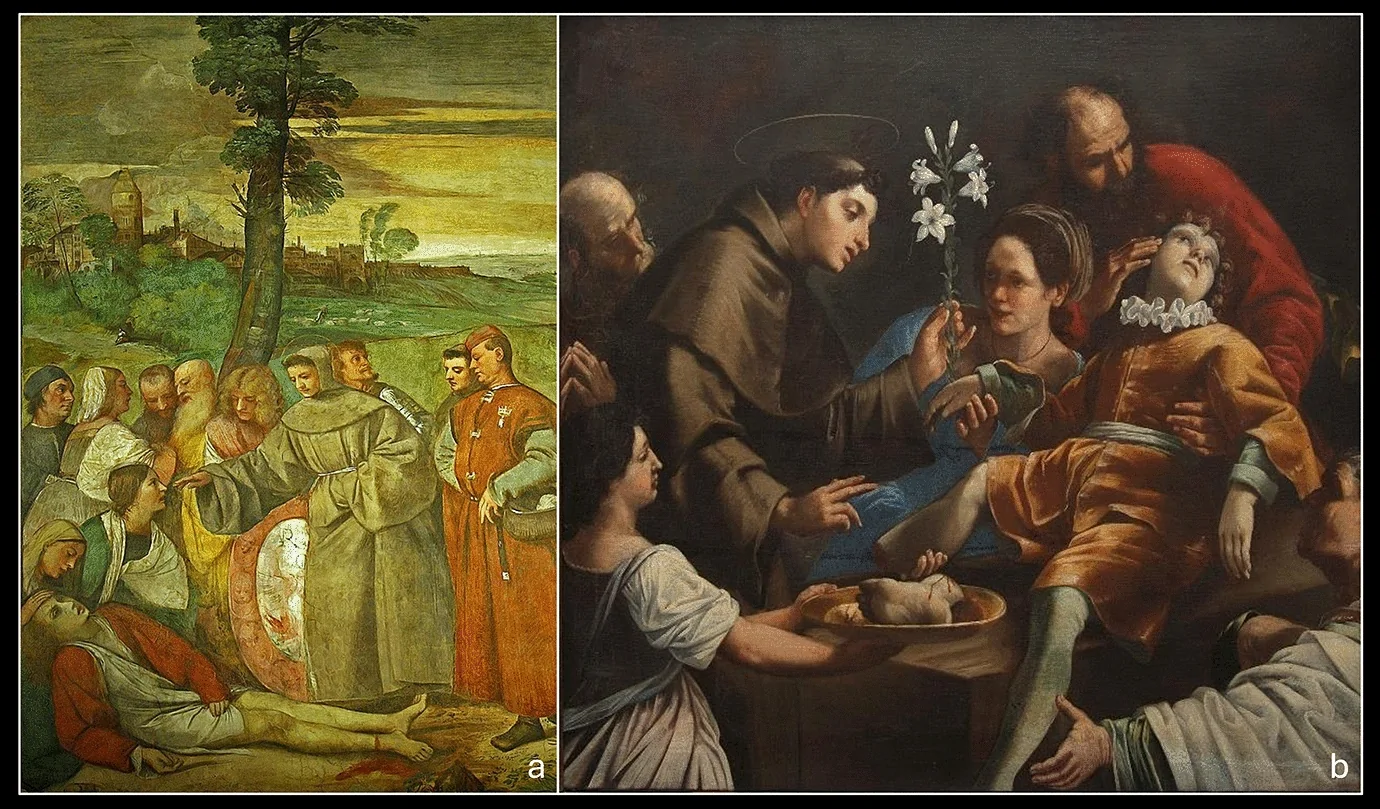
**a** Titian Vecellio, *The Reattachment of the Severed Foot by Saint Anthony of Padua*, 1511, Scoletta del Santo, Basilica of Saint Anthony, Padua, Italy. **b** Antonio Giarola, *The Reattachment of the Severed Foot by Saint Anthony of Padua*, 1630–1636, Church of Saint Fermo, Verona, Italy

In the sixteenth century, the scene was reinterpreted by Tullio Lombardo (b. in 1460—d. on 17 November 1532 in Venice, Italy) in one of nine marble bas-reliefs adorning the Ark of the Saint completed in 1504 (Fig. 6b); by Titian Vecellio (b. 1485 in Pieve di Cadore—d. 27 August 1576 in Venice) in one of eighteen painted panels on the walls of the Scoletta del Santo (Fig. 7a), thirteen of which depict the Miracles of the Saint (1511); by Girolamo Tessari, known as Dal Santo (b. ca. 1490—d. in 1561 in Padua, Italy), in a scene painted in 1535 at the wall of the *Santuario del Noce in Camposampiero*, where the Saint was living before his death (Fig. 5b) (Guarnieri, 2021; Wilk, 1983). Inspired by Donatello, Tullio Lombardo and Titian, who had the privilege of working in the Basilica of Saint Anthony, many artists were commissioned to represent this miraculous healing until the eighteenth century, among them: Antonio Giarola (b. in Verona, ca. 1597—d. ca. 1665) in the church of Saint Fermo in Verona (1630–1636) (Fig. 7b), *Pietro Ricchi* o Righi, detto il Lucchese (b. in Lucca in 1606—d. in Udine in 1675) in the church of Saint Francis in Lucca and Giambattista Tiepolo (b. in Venezia in 1696—d. in Madrid in 1770) in the church of Saint Michael the Archangel in Mirano. Therefore, the commission of the confrères of the *Veneranda Arca del Santo* became an *Exemplum* because it portrays, with realistic precision, the moment of the foot’s healing, which is conveyed through the patient’s suffering and shared emotionally by the bystanders (Puppi, 1979). The physical pain depicted in these works not only symbolises the suffering of the soul necessary for attaining salvation but is also realistically expressed through the positioning of the body’s limbs and the contortions of the face. The decision to highlight this miraculous action, attributed explicitly to Saint Anthony, the quintessential miracle worker, and to repeat its depiction in several key locations within the Antonian domain, is closely tied to the dedication of a church to Saint Anthony, placed under the direct authority of the Holy See of Rome. Padua, a renowned university city since 1222 and home of the Collegium of Medical Doctors and Artists from ca. 1250, was considered the most fitting location to display and glorify the Saint’s relics and miracles, particularly the replantation of the foot. This was especially significant because Anthony, who suffered from gout, found it difficult to move while preaching, and towards the end of his life, chose to live in the branches of a walnut tree to avoid the pain caused by his illness (Major, 2019). Hence, the Saint’s illness and subsequent death take on deep symbolic meaning, as his personal sacrifice seems to grant him the miraculous power to heal his followers.

The masterpieces of Donatello and Tullio Lombardo (Fig. 6a and b) showed in the Basilica influenced the scenes frescoed by Titian on the walls of the “Scoletta”, a confraternity dedicated to Saint Anthony, located adjacent to the great Basilica, on commission of the *Massari dell’Arca*, the governing body of the Basilica itself (Fig. 7a). Titian painted two other miracles in addition to the *Miracle of the Reattached Leg*: *The Miracle of the Newborn Child* and the *Miracle of the Jealous Husband*. These pieces represent his first securely documented works and his initial independent commissions following the death of Giorgione, with whom Titian began his career, painting alongside other artists, at the *Fondaco dei Tedeschi* in Venice. Even if Titian had taken inspiration by the classicism of the Donatello’s and Tullio’s scenes, whose artworks are melodramatic but quite far from the watcher, he try to find here, for the first time in his career, a new type of classicism, more realistic and controlled at the same time, which must be recognisable by his clients, suitable both for private and public devotion. With Titian, feeling is expressed more subtly, as seen in the mother’s meaningful glance as she implores Saint Anthony to heal her son’s leg.

## The Miracles of St Thomas of Hereford

Thomas de Cantilupe, Bishop of Hereford, was born around 1218 in Buckinghamshire, England, into a noble family with Norman roots. His father held a position in the royal household, and one of his uncles was the Bishop of Worcester (Bass, 2020; Finucane, 1995). From a young age, Thomas was destined for the church, studying at Oxford, Paris and Orleans, with a focus on civil and canon law. He became Chancellor of Oxford University in 1261 and later joined the baronial party led by Simon de Montfort. After the defeat at the Battle of Evesham, Thomas studied theology in Paris and regained the king’s favour in 1266, returning to England. He became the Bishop of Hereford in 1275, where he was known for his devout and austere lifestyle (Clarke, 1997). He died in 1282 while travelling to Orvieto, possibly from malaria. His bones were later moved to Hereford Cathedral, where his relics became associated with numerous miracles. In 1307, investigations into these miracles began as part of his canonisation process, and he was officially canonised in 1320 (Bannister, 1905; A Companion to Medieval Canonization Processes, ed. Brill, 2025).

One of the most widely recounted miracles attributed to Thomas de Cantilupe involves the remarkable recovery of a young girl named Alice, whose case was documented in early 14th-century England. In 1293, William Lowther, Earl of Lonsdale, embarked on a journey from northern England to London, accompanied by his five-year-old daughter, Alice (Bartlett, 2006; Kemp, 1948). He was en route to Spain to fulfil a religious vow to visit the shrine of St. James at Santiago de Compostela. During their passage near Stamford, Alice sustained a traumatic injury to her right foot after tripping into a hole in the road, resulting in multiple fractures (Vauchez, 1997). William continued the journey, carrying her on his shoulders, and eventually reached London. They crossed London Bridge and took temporary lodging in Southwark. Shortly after their arrival, Alice developed a progressive illness. She experienced a sudden onset of weakness and loss of sensation in the left side of her body, with preserved function limited to her right hand. The debilitating nature of her condition forced father and daughter into destitution. Alice, now unable to walk, resorted to dragging herself along by her arms, and they survived by begging in doorways. Her previously fractured foot deteriorated significantly, showing signs of advanced infection with visible bone protrusions and purulent discharge (Webster, 2015).

Alice later reported, during an inquiry in 1307, that she had attempted to cleanse the infected limb using water from the well at the Church of St. Clement Danes, her only recourse as a beggar. She also received rudimentary medical attention from a local surgeon, Master Gilbert, who applied topical treatments but declared the condition incurable (Hanska, 2001).

William had likely heard of the posthumous reputation for healing associated with Thomas de Cantilupe, the former Bishop of Hereford, whose cult was gaining recognition in London. He prayed for his daughter’s recovery, placing a coin on her head as a votive offering and vowing to undertake a pilgrimage to Cantilupe’s tomb in Hereford, pledging to dedicate Alice to the Virgin Mary if she were healed.

He transported her in a rudimentary one-wheeled cart, likely a wheelbarrow, and they arrived at Hereford on the night of Pentecost in 1303. While staying at the shrine, Alice experienced a vision in which an elderly man, clothed in luminous white garments and wearing a richly adorned headdress, appeared to her. He reportedly anointed her body with a milky substance from a small container, tracing a path from her chest to her afflicted limbs, including the ulcerated right foot and the paralysed left leg (Daly, 1982).

Following this vision, Alice’s condition began to improve. The discharging ulcers on her foot dried up, and she regained the ability to walk, albeit with a limp and requiring a walking stick due to contractures and scarring, particularly in the tendons of the right foot. Within a week, her physical function had significantly improved (Webb, 1996; Cambridge Univ. Occasional Paper (Year)).

## The Replanting of the Foot by Saint Peter Martyr

The miracle of the replanting of a severed foot is famously attributed to Saint Peter Martyr in the frescoes painted in 1468 by Vincenzo Foppa (b. ca. 1420—d. in 1516 in Brescia, Italy) in the Portinari Chapel in the Church of Saint Eustorgio in Milano. Also known as Peter of Verona (b. in 1205 as Pietro Rosini in Verona, Italy—d. in 1252 in Monza, Italy), he was an Italian Dominican friar and inquisitor and is best known for his tireless defence of the Catholic faith against the Cathar heresy and for the dramatic circumstances of his death. These circumstances shaped both his iconographic attributes and the popular veneration that followed. Among the most frequently represented saints in Western art, Peter’s visual prominence stems mainly from his violent martyrdom. Saint Peter Martyr was canonised in 1253 by Pope Innocent IV, just one year after his death (one of the fastest canonisations in Church history). His feast day is April 29 (formerly April 6 on the Dominican calendar) (ChristianIconography.info. “St Peter Martyr Restores a Severed Limb”).

Born into a family sympathetic to Catharism, Peter embraced the Catholic faith early in life. By the age of seven, he had learned to recite the Nicene Creed. He later enrolled at the University of Bologna, where, at the age of fifteen, he met Saint Dominic de Guzmán (b. 8 August 1170 in Spain—d. 6 August 1221 in Bologna, Italy), founder of the Order of Preachers. According to the *Martyrology*, *“Peter received the habit from Saint Dominic himself”*. Peter quickly distinguished himself as a gifted preacher, tasked by the Holy See with combating heresy across central and northern Italy, from Rome to Milan. In 1244, he was sent to Florence, where his sermons brought countless individuals back to the Catholic faith. His preaching so impressed the seven founding saints of the Servite Order that Peter became their spiritual father. After intense prayer, he received confirmation from the Blessed Virgin Mary herself that their foundation was of divine origin (Chiostri di Sant’Eustorgio official site). His reputation as an uncompromising defender of orthodoxy earned him admiration from many, as well as animosity from heretical groups. His ascetic lifestyle and exceptional familiarity with Scripture only added to his mystique (ChristianIconography.info, Image descriptions confirming the healed foot scene among Foppa’s frescoes). In 1251, Pope Innocent IV appointed him inquisitor for Lombardy, a position that put him in direct conflict with entrenched Cathar communities. Undaunted by the dangers of his mission, Peter openly expressed his desire for martyrdom. According to a companion, he would pray for this grace each time he elevated the chalice during Mass. His wish was fulfilled on 6 April 1252, when he was ambushed on the road from Como to Milan by a Cathar hired to kill him. The assassin struck Peter’s skull with an axe, mortally wounded his companion Domenico and then stabbed Peter in the chest with a dagger. Before dying, the saint dipped his finger in his own blood and traced the words *Credo in unum Deum (“I believe in one God”)* on the ground. His body was brought to Milan and enshrined in the Basilica of Saint Eustorgio, where, since 1340, it has rested in the splendid *Ark of Saint Peter Martyr*, a Gothic masterpiece sculpted by Giovanni di Balduccio (b. in 1290, Pisa, Italy—d. in 1365). The popular veneration that followed was swift and intense, bolstered by numerous miracles attributed to Peter’s intercession. He was canonised just eleven months after his death.

**Fig. 8:**
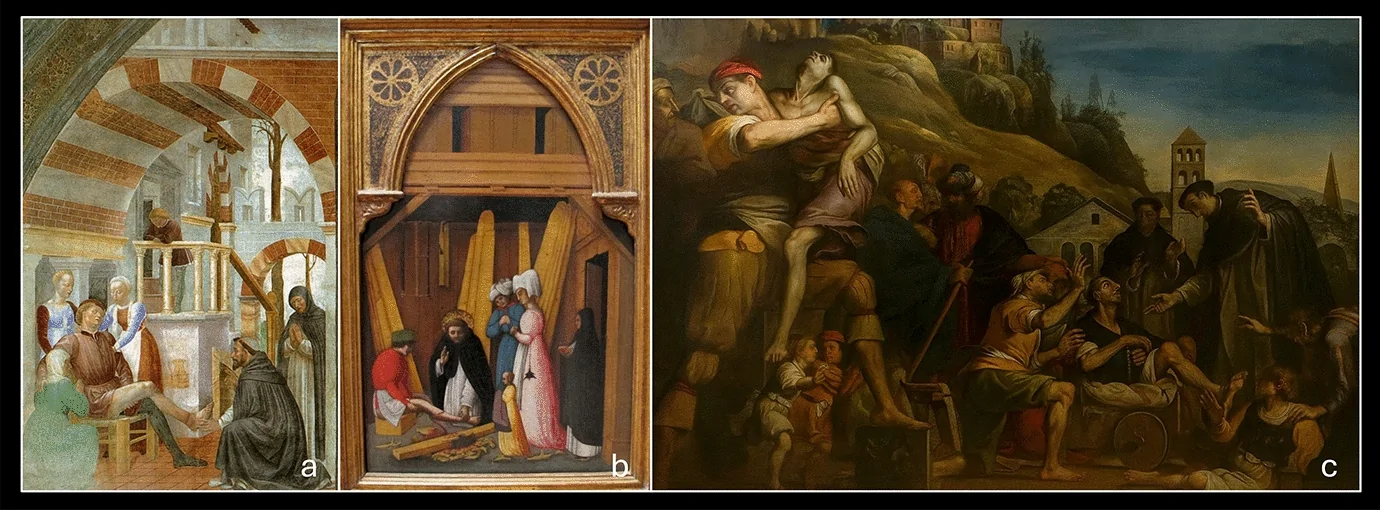
**a** Vincenzo Foppa, *The Healing of the Foot by Saint Peter Martyr*, 1468, Portinari Chapel, Church of Saint Eustorgio, Milan, Italy. **b** Antonio Vivarini, *The Healing of the Leg by Saint Peter Martyr* (detail from the Polyptych of Saint Peter Martyr), ca. 1450–1460, The Metropolitan Museum of Art, New York, USA. **c** Giovanni Paolo Ghianda, *The Healing of the Foot by Saint Peter Martyr*, 1629, Civic Art Gallery, Como, Italy

Saint Peter of Verona is immediately recognisable in religious art by the symbols of his martyrdom: the Dominican habit and the gaping wound or weapon embedded in his skull. Many images of Peter feature miracles attributed to his intercession, both during his lifetime and posthumously. One of the most famous is the miracle of the severed foot (Jacopo da Varagine, 1900a, 1900b). This episode appears in one of the two primary narrative cycles housed in the Church of Saint Eustorgius, Milan, Italy, where Peter’s relics lie. Among the eight sculpted panels of the Ark that narrate the life, martyrdom and the rise of the cult, the healing of the invalid is only generically referable to the severed foot. In contrast, the series of frescoes painted in 1468 by Vincenzo Foppa (b. in 1430—d. in 1515 in Brescia, Italy), depicting the saint performing various miracles, such as exposing a fraudulent vision, miraculously cooling a summer crowd with a cloud and writing the Creed in the dirt as he died, includes the miraculous healing of a severed foot being reattached (Fig. 8a). This scene is rendered with a realistic setting and compelling expressions on both the patient and the healer saint.

Although most images portray Peter as a man in the prime of life, some show him younger, while others portray him with a grey beard. He is often depicted alongside other Dominicans in their churches, underscoring his central role in the Order’s history and spiritual identity. The iconography of foot reattachment is well represented in the fifteenth-century Altarpiece of Saint Peter Martyr in t*he Capuchin monastery* in Parma, where in one of the scenes of his life, Bartolomeo e Agnolo degli Erri painted, in vivid detail, the amputation and subsequent miraculous reattachment of a foot performed by the saintly healer (Met Museum: Antonio Vivarini’s painting Saint Peter Martyr Healing the Leg of a Young Man).

This event reflects the deep connection between the saint’s cult and miraculous healings. This link took firm root from medieval iconography, as shown by many masterpieces from the fifteenth century onward, which depict the same miracle: in 1450–60 by Antonio Vivarini (also called Antonio of Murano active ca. 1440–1480) for the polyptych of Saint Peter Martyr (Fig. 8b); in 1629 by Giovanni Paolo Ghianda (b. in 1593 in Como—d. in 1637 in Como) for the church of Saint John Pedemonte in Como (Fig. 8c); in 1637 by Giovanni Mauro Della Rovere Detto Fiamminghino (b. in Milan 1575—d. in Milan 1640) for the church of Saint Peter Martyr in Novara; in ca. 1677 by Giovanni Battista Costa (active in Milan in the seventeenth century) for the private chapel of Palazzo Arese Borromeo (Monza Brianza) (https://www.lombardiabeniculturali.it/blog/percorsi/ville-di-delizia-briantee-poste-sulla-tratta-delle-ferrovie-nord-milano/palazzo-arese-borromeo-a-cesano-maderno); in around 1710–1720 by Giovanni Battista Cozza (b. in Milan 1676—d. in Ferrara 1742) for the church of Saint Dominic in Ferrara.

## Saint Ignatius of Loyola’s Conversion After His Leg and Foot Injuries

**Fig. 9:**
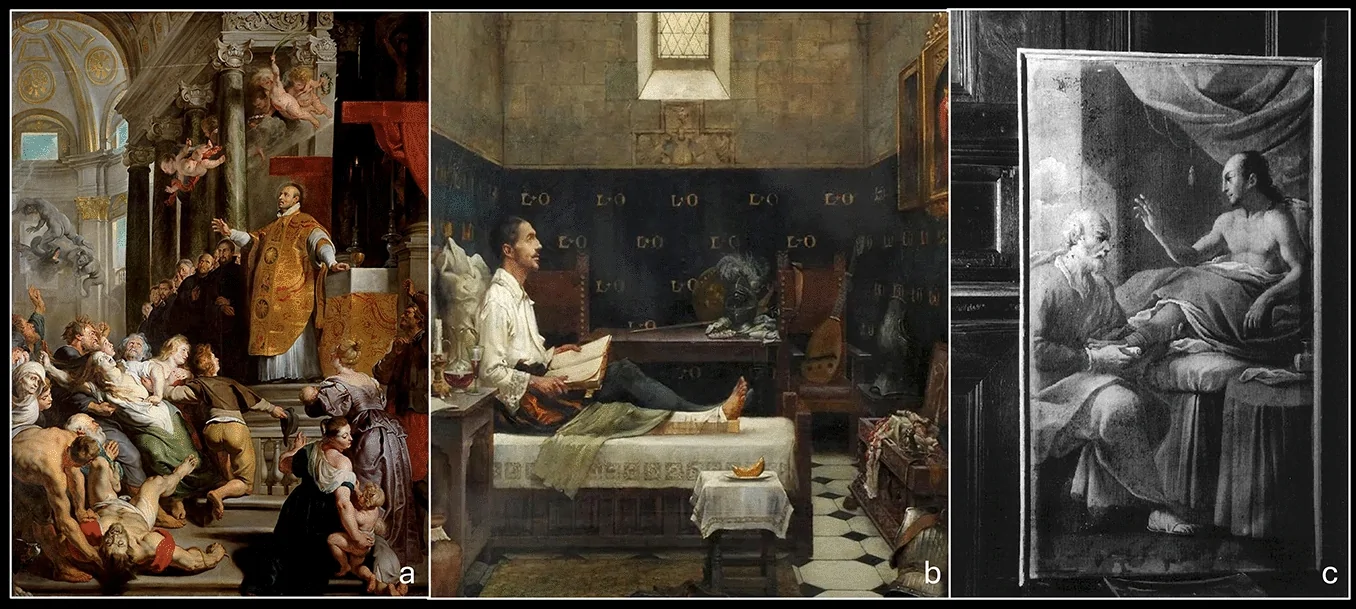
**a** Peter Paul Rubens, *The Miracles of Saint Ignatius of Loyola*, 1615–1616, Kunsthistorisches Museum, Vienna, Austria. **b** Albert Chevallier Tayler, *The Healing and Conversion of Ignatius through the Intercession of Apostle Peter*, 1904, Chapel of Saint Ignatius, Church of the Sacred Heart, Wimbledon, UK. **c** Giovanni Evangelista Draghi (Piacentino), *The Healing and Conversion of Ignatius through the Intercession of Apostle Peter*, 1686, Church of Saint Ignatius of Loyola, Busseto (Parma), Italy

Saint Ignatius of Loyola (b. in 1491, in Loyola, Spain—d. in 1556, Rome, Italy) is the patron saint of trauma and fracture surgery, and his feast day is celebrated on July 31st. He is not primarily known for performing miracles in the traditional sense (such as healing the sick or supernatural acts), but rather for his spiritual influence, transformative teachings and founding of the Jesuits, which had a powerful and lasting effect on the Church and the world. However, after his death, many people prayed for his intercession, and several miracles were reported, especially healings, which contributed to his canonisation in 1622 (Limpo Trigueiros et al., 2022). The Church formally investigated these miraculous healings as part of the canonisation process. Some of them were depicted by the Flemish artist Peter Paul Rubens (b. in Siegen in 1577—d. in Antwerp in 1640), in the renowned work *The Miracles of Saint Ignatius of Loyola,* dated 1615–1616 (Fig. 9a) (Vlieghe, 1990). In this quintessential example of the Baroque era, three miracles have been depicted. On the left side, a possessed woman violently throws back her head, pulling at her long, loose hair as she is held by the central figure of a man with his back to the viewer. His bare, dirty foot is closest to the viewer, a stark reminder of the shared earthly realm, contrasting with the hint of celestial light at the top of the painting. The second miracle, to the right, involves the old washer woman whose withered arm has been restored after she washed the saint’s linens. She holds up the cloth as a testament and as a gesture of gratitude. Thirdly, the figures of women, children and babies serve as a reference to the saint’s role as intercessor in difficult births. In the foreground on the right, Rubens depicts a young mother, seemingly amazed, as her infant comes to life lying on the ground before her.

Ignatius of Loyola was born in 1491 in Azpeitia, in the Basque region of Spain, as Íñigo López de Loyola. He was the youngest of thirteen children in a noble family. Although initially destined for a clerical career, he chose the military path instead, serving as a page and then as a knight at the court of Don Juan Velázquez de Cuéllar, treasurer to King Ferdinand the Catholic (Saint Ignatius of Loyola, 2014a, 2014b).

Saint Ignatius of Loyola spent his early years as a knight in the service of Antonio Manrique de Lara, 2nd Duke of Nájera. On 20 May 1521, during the defence of Pamplona against French forces, he sustained a catastrophic cannonball injury that resulted in bilateral femoral fractures. Although he survived the acute trauma, he was left with a significant leg length discrepancy and a visibly deformed limb (Rosenman, 1996). Dissatisfied with the cosmetic and functional outcome, he elected to undergo further procedures aimed at “re-setting” the fractures (Bartoli, 1650; Saint Ignatius of Loyola, 2014a, 2014b). These included a prolonged period of skeletal traction, during which a cannonball was used as a counterweight to achieve anatomical realignment, as illustrated in one of the religious paintings by Albert Chevallier Tayler (b. in Leytonstone in 1862—d. in London in 1925) (Fig. 9b) (Saint Ignatius of Loyola, 2014a, 2014b).

Ignatius underwent multiple painful surgeries to correct a poorly healed leg injury, including breaking the bone again and removing a protruding bone without anaesthesia. Determined to maintain his appearance for high society, he endured the intense pain in silence. His treatment, which included early forms of traction and bone realignment, reflects the primitive orthopaedic methods of the time. His extraordinary endurance marked the start of a profound spiritual transformation. Saint Ignatius of Loyola underwent what is now recognised as an early osteotomy: his flesh was cut open, and a protruding bone was sawed off to correct a leg deformity (Giornale Italiano di Ortopedia e Traumatologia, 2020). Doctors used ointments and prolonged traction to prevent leg shortening, causing days of intense pain. Pietro Panzeri, founder of Italy’s first orthopaedic surgeons’ association, cited this case in his 1881 work Annotations on Orthomorphic Surgery, naming Ignatius as the first documented osteotomy patient, though without elaborating on the details (Panzeri, 1881; Vandenbulcke et al., 2025). At one point, his condition worsened dramatically. He couldn’t eat and showed signs believed to precede death. On the feast of Saint John, doctors gave up hope, and he received the sacraments. But on the eve of Saints Peter and Paul, he began to recover, an event Ignatius later saw as a miracle due to his devotion to Saint Peter.

Despite these interventions, the injuries left him unfit for military service. During his long convalescence, with access to only two books: one recounting the life of Christ and the other detailing the lives of the saints, Ignatius underwent a profound spiritual transformation. Inspired by these readings, he abandoned his military ambitions and eventually became a priest, founding the Society of Jesus (Jesuits) (Limpo et al., 2022).

Most representations of the healing of the injured leg are dated after or shortly before the canonisation of the Saint, in 1622. Among them is *The healing and apparition of Saint Peter and the Virgin*, painted by Domingos da Cunha (ca. 1598, Lisbon—11 May 1644, Lisbon), along with other episodes from the life of Saint Ignatius in the *New Cathedral in Coimbra*, ca. 1640. Some decades after, in 1686, in one of the various churches dedicated to Saint Ignatius of Loyola in Busseto in the province of Parma, the Farnese’s painter Giovanni Evangelista Draghi called Piacentino (1654, Genova; 1712, Piacenza) painted six canvas with *Episodes of the Saint’s life*; one of them is the *Healing and the resulting Conversion by Ignatius through the intercession of Apostle Peter* (Fig. 9c) (Braga, 1977).

## The Healing of Lower Limbs Through the Intercession of Saint Charles Borromeo

The figure of the physician Saint Carlo Borromeo (b. 2 October 1538 in Arona, Italy—d. 3 November 1584 in Milan, Italy), to whom are attributed various healings, became fundamental after the second half of the sixteenth century during the Catholic-Reformation (Brivio, 1610). Patron saint of bishops, seminarians and catechists, he is remembered for his deep humility, reformist zeal and tireless service to the Church (Giussani, 1610).

Borromeo was a descendant of nobility, and his family was one of the most ancient and wealthy in Lombardy. On 6 December 1559, at the age of 21, he earned a doctorate in canon and civil law from the University of Pavia. On 25 December 1559, Borromeo was summoned to Rome by his uncle Cardinal Giovanni Angelo Medici, who was elected Pope Pius IV. During his four years in Rome, Borromeo lived in austerity, obliged the Roman Curia to wear black, and established the Academy of the Vatican Knights, an academy of learned persons, publishing their memoirs as the Noctes Vaticanae (Brivio, & Gregori, 1999).

On 31 January 1560, Charles Borromeo was appointed Cardinal and Archbishop of Milan by his uncle, Pope Pius IV, despite not yet being ordained as a priest (Fappani, 1984). Borromeo was appointed the administrator of the Archdiocese of Milan on 7 February 1560. Following his ordination, he took an active role in implementing the reforms of the Council of Trent within his diocese. He played a key role in organising the Council itself (1545–1563), the last session 1562–1563, which sought to address the challenges of the Protestant Reformation and to renew the Catholic Church from within (Jedin, 1957). Throughout his life, Cardinal Borromeo was deeply committed to raising the standards of the clergy. He founded seminaries and colleges, emphasised moral discipline and encouraged a stronger sense of pastoral responsibility among priests and bishops (O’Malley, 2013).

During the devastating plague that struck Milan between 1576 and 1578, Borromeo chose to remain in the city, tirelessly caring for the sick. He organised relief efforts, led public prayers and even sold church treasures to provide aid to the suffering population. Exhausted by years of self-sacrifice and relentless work, he died on 3 November 1584 at the age of 46 (Uffizi Galleries, 2020). He was canonised by Pope Paul V in 1610, and his feast day is celebrated on November 4.

In 1602, twenty-six years after Charles’ death, the first cycle of Quadroni was begun in Milan, known as *“The Facts of the Life of Blessed Charles”*, and consisting of 28 paintings depicting his life, with a focus on his tenure as Archbishop of Milan (Lombardia Beni Culturali, 2010).

The second cycle of the Quadroni consists of 24 large paintings, smaller than those in the first set, and depicts the Miracles of St. Charles. These works were painted between December 1609 and 1 November 1610, coinciding with the canonisation of St. Charles. The paintings were first displayed alongside the initial cycle on 4 November 1610, in the nave of the Cathedral of Milan and subsequently exhibited every year throughout November to commemorate the feast of Saint Charles on the same date. In keeping with the statutes of the Council of Trent, this art was conceived as a clear and didactic language, intended to inspire the faithful towards piety and devotion (Gregori, 1999). The healings represented in the Quadroni, most of them of lower limbs, are carried out after the death of Saint Charles Borromeo by friar-medics-healers acting in his name and through his saving intercession. The patients are anonymous people, except a few who referred to recognisable people: *Aurelia degli Angeli*, painted by Giovanni Battista Crespi known as il Cerano (Romagnano Sesia 1573—Milan 1632), the crippled baby *Giovanna Marone* by Cerano (Treccani, 1931) and the paralytic *Gerolamo Baio* by Giulio Cesare Procaccini (b. 30 May 1574 in Bologna, Italy—d. 14 November 1625 in Milan, Italy) (Treccani, 2016; Brivio, 1995).

Cerano was a friend of Cardinal Federico Borromeo and one of the official painters of the Quadroni, in which he interpreted the statutes of the Council by painting his realistic characters with full expression (Bezalel, 2010).

**Fig. 10:**
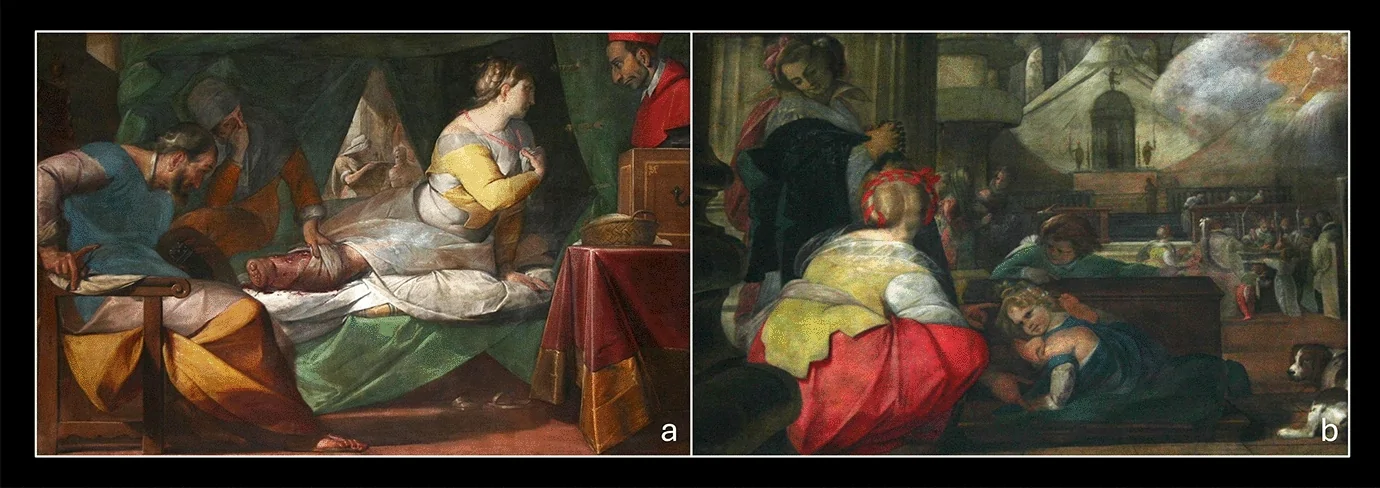
**a** Giovanni Battista Crespi (Cerano), *The Healing of Aurelia degli Angeli*, ca. 1610, Milan Cathedral, Italy. **b** Giovanni Battista Crespi (Cerano), *The Healing of the Crippled Infant Giovanna Marone through the Intercession of Saint Charles Borromeo*, ca. 1610, Milan Cathedral, Italy

Aurelia degli Angeli is depicted as a young woman suffering from erysipelas, a disease caused by germs like *Streptococcus* or *Staphylococcus Aureus*, evident by the holes in the skin where rotten flesh is clearly visible. Aurelia holds her hands to her chest in a sign of devotion and looks at the face of Saint Charles, who is looking at her benevolently on the right. The surgeon, on the left, is amazed, and the servant, near to him, tries to protect herself from the strong smell of the infection (Fig. 10a) (Centro Studi GISED, 2010).

The healing of the crippled baby Giovanna Marone, affected by a particular lower limb malformation so that she appears like a contortionist over the altar in the painting by Cerano and where her mother prays for her healing, strikes the imagination of people so that the painter Antonio De Bellis (active in Naples from ca. 1630–1660) represents the same subject few years later in 1636–1638 (Fig. 10b). The most impossible miraculous events appeared to be the healing of Aurelia and of the baby Giovanna Marone, the former because of the very severe infection and the second because of the very devastating malformation. The most valuable was the intervention of the holy doctors thanks to the saving intercession of Saint Charles (Duomo di Milano, 2020).

## Conclusion

This study highlights the frequent use of works of art from the thirteenth century onward to depict the miraculous healing of the foot and leg. These images illustrated how individuals suffering from various injuries or pathologies were healed by divine figures such as Jesus Christ, the Virgin Mary, the Apostles or through the intercession of Saints, particularly the Holy Healers. The role of the Holy Healer Doctor was instrumental in the establishment of the first hospitals, often built adjacent to churches and monasteries. This role remained highly esteemed until the mid-eighteenth century, when the Order of Physicians and Surgeons of Venice compiled a list of the most renowned Holy Healer Doctors.

With the scientific revolution initiated by Andreas Vesalius, who collaborated with artists like Jan Stephan von Calcar (b. in 1499 in Calcar, Cleve—d. in 1546 in Naples, Italy), doctors and artists began working closely together. Both Vesalius and Titian served at the court of Charles V, King of Spain and Holy Roman Emperor, further bridging the gap between art and medicine. In the sixteenth century, Girolamo Fabrici d’Acquapendente, a pioneer in anatomy, collaborated with the artist Dario Varotari (b. in 1539 in Verona, Italy—d. in 1569 in Padua, Italy) and his son Alessandro said the *Padovanino* (b. in 1588 in Padua, Italy—d. in 1649 in Venice, Italy)*,* who not only painted the* “Pitture Colorate d’Anatomia”*, but also built and decorated his Villa near Padova, in Mandria (Marcon, 2004).

This intersection of religion, art and medicine, as explored in this study, explains why many of the first hospitals were built near churches and convents. In Florence, the Hospital of the Innocents was built starting in 1417 by the famous architect Filippo Brunelleschi (b. in 1377—d. on 15 April 1446 in Florence, Italy) near the ancient Church of the Most Holy Annunciation, founded by Matilde di Canossa and restored from 1444 by Michelozzo. In Padua, the hospital of Saint Francis was established, near the church and convent of Saint Francis, from 1414 by Sibilla de Cetto (b. in 1350—d. in 1421 in Padua, Italy), linked to the Carrara family, under the supervision of Galvano of Bartolomeo Lattuga, the same master foreman who was in charge of the restoration of the Basilica of Saint Anthony of Padua.

No longer relying solely on the miracles of the Holy Healer Doctor, Vesalius interpreted the new healing practices of emerging physicians as evidence of divine presence: *“The full knowledge of human nature shows the wisdom of the Creator”.* This shift in perspective allowed religion and sacred art to pave the way for the development of modern medical sciences, including the field that would one day become orthopaedics.

## Data Availability

No datasets were generated or analysed during the current study.
